# Supplementary material for: Association between the Quantity of Nurse–Doctor Interprofessional Collaboration and in-Patient Mortality: A Systematic Review
Source: Int J Environ Res Public Health. 2024 Apr 17;21(4):494. doi: 10.3390/ijerph21040494 (PMC11050129; doi:10.3390/ijerph21040494)
Supplement: Supplementary file 1 [file ijerph-21-00494-s001.zip › Supplementary material S2_Search Strategy.pdf]

MEDLINE

S. No.	Searches
1	Patient Care Team/ or intersectoral collaboration/ or interprofessional relations/ or interdisciplinary communication/ or Physician-Nurse Relations/
2	((Interprofessional or "Inter professional" or Intersectoral or interdisciplinary or "inter disciplinary") adj3 (collaborat* or co-operat* or cooperat* or communicat* or interact* or relation* or discussion* or round* or meeting or teamwork or care)).ti,ab.
3	((Multiprofessional or "multi professional" or multidisciplinary or "multi disciplinary") adj3 (collaborat* or co-operat* or cooperat* or communicat* or interact* or relation* or discussion* or round* or meeting or teamwork or care)).ti,ab.
4	((crossdisciplinary or "cross disciplinary") adj3 (collaborat* or co-operat* or cooperat* or communicat* or interact* or relation* or discussion* or round* or meeting or teamwork or care)).ti,ab.
5	((transdisciplinary or "trans disciplinary") adj3 (collaborat* or co-operat* or cooperat* or communicat* or interact* or relation* or discussion* or round* or meeting or teamwork or care)).ti,ab.
6	((Interoccupation* or "inter occupation" or "inter occupational") adj3 (collaborat* or co-operat* or cooperat* or communicat* or interact* or relation* or discussion* or round* or meeting or teamwork or care)).ti,ab.
7	(("doctor nurse" or "physician nurse") adj3 (collaborat* or co-operat* or cooperat* or communicat* or interact* or relation* or discussion* or round* or meeting or teamwork or care)).ti,ab.
8	1 or 2 or 3 or 4 or 5 or 6 or 7
9	Mortality/ or death/ or hospital mortality/
10	(death* or mortalit* or fatalit*).ti,ab.
11	9 or 10
12	8 and 11

EMBASE

S. No.	Searches
1	doctor nurse relation/ or intersectoral collaboration/ or interdisciplinary communication/ or collaborative care team/ or patient care planning/
2	((Interprofessional or "Inter professional" or Intersectoral or interdisciplinary or "inter disciplinary") adj3 (collaborat* or co-operat* or cooperat* or communicat* or interact* or relation* or discussion* or round* or meeting or teamwork or care)).ti,ab.
3	((Multiprofessional or "multi professional" or multidisciplinary or "multi disciplinary") adj3 (collaborat* or co-operat* or cooperat* or communicat* or interact* or relation* or discussion* or round* or meeting or teamwork or care)).ti,ab.
4	((crossdisciplinary or "cross disciplinary") adj3 (collaborat* or co-operat* or cooperat* or communicat* or interact* or relation* or discussion* or round* or meeting or teamwork or care)).ti,ab.
5	((transdisciplinary or "trans disciplinary") adj3 (collaborat* or co-operat* or cooperat* or communicat* or interact* or relation* or discussion* or round* or meeting or teamwork or care)).ti,ab.
6	((Interoccupation* or "inter occupation" or "inter occupational") adj3 (collaborat* or co-operat* or cooperat* or communicat* or interact* or relation* or discussion* or round* or meeting or teamwork or care)).ti,ab.
7	(("doctor nurse" or "physician nurse") adj3 (collaborat* or co-operat* or cooperat* or communicat* or interact* or relation* or discussion* or round* or meeting or teamwork or care)).ti,ab.
8	1 or 2 or 3 or 4 or 5 or 6 or 7
9	mortality/ or death/ or mortality rate/ or hospital mortality/
10	(death* or mortalit* or fatalit*).ti,ab.
11	9 or 10
12	8 and 11

PsycINFO

S. No.	Searches
1	interdisciplinary treatment approach/ or interpersonal interaction/ or collaboration/
2	((Interprofessional or "Inter professional" or Intersectoral or interdisciplinary or "inter disciplinary") adj3 (collaborat* or co-operat* or cooperat* or communicat* or interact* or relation* or discussion* or round* or meeting or teamwork or care)).ti,ab.
3	((Multiprofessional or "multi professional" or multidisciplinary or "multi disciplinary") adj3 (collaborat* or co-operat* or cooperat* or communicat* or interact* or relation* or discussion* or round* or meeting or teamwork or care)).ti,ab.
4	((crossdisciplinary or "cross disciplinary") adj3 (collaborat* or co-operat* or cooperat* or communicat* or interact* or relation* or discussion* or round* or meeting or teamwork or care)).ti,ab.
5	((transdisciplinary or "trans disciplinary") adj3 (collaborat* or co-operat* or cooperat* or communicat* or interact* or relation* or discussion* or round* or meeting or teamwork or care)).ti,ab.
6	((Interoccupation* or "inter occupation" or "inter occupational") adj3 (collaborat* or co-operat* or cooperat* or communicat* or interact* or relation* or discussion* or round* or meeting or teamwork or care)).ti,ab.
7	(("doctor nurse" or "physician nurse") adj3 (collaborat* or co-operat* or cooperat* or communicat* or interact* or relation* or discussion* or round* or meeting or teamwork or care)).ti,ab.
8	1 or 2 or 3 or 4 or 5 or 6 or 7
9	mortality rate/
10	(death* or mortalit* or fatalit*).ti,ab.
11	9 or 10
12	8 and 11

CINAHL Search Strategy

S. No.	Search Terms
S1	(MH "Multidisciplinary Care Team") or (MH "Interprofessional Relations") or (MH "Nurse-Physician Relations")
S2	TI ((Interprofessional or "Inter professional" or Intersectoral or interdisciplinary or "inter disciplinary") N3 (collaborat* OR co-operat* OR cooperat* OR communicat* OR interact* OR relation* OR discussion* OR round* OR meeting OR teamwork OR care))
S3	AB ((Interprofessional or "Inter professional" or Intersectoral or interdisciplinary or "inter disciplinary") N3 (collaborat* OR co-operat* OR cooperat* OR communicat* OR interact* OR relation* OR discussion* OR round* OR meeting OR teamwork OR care))
S4	TI (Multiprofessional or "multi professional" or multidisciplinary or "multi disciplinary") N3 (collaborat* OR co-operat* OR cooperat* OR communicat* OR interact* OR relation* OR discussion* OR round* OR meeting OR teamwork OR care))
S5	AB (Multiprofessional or "multi professional" or multidisciplinary or "multi disciplinary") N3 (collaborat* OR co-operat* OR cooperat* OR communicat* OR interact* OR relation* OR discussion* OR round* OR meeting OR teamwork OR care))
S6	TI ((crossdisciplinary or "cross disciplinary") N3 (collaborat* OR co-operat* OR cooperat* OR communicat* OR interact* OR relation* OR discussion* OR round* OR meeting OR teamwork OR care))
S7	AB ((crossdisciplinary or "cross disciplinary") N3 (collaborat* OR co-operat* OR cooperat* OR communicat* OR interact* OR relation* OR discussion* OR round* OR meeting OR teamwork OR care))
S8	TI ((Interoccupation* or "inter occupation" or "inter occupational") N3 (collaborat* OR co-operat* OR cooperat* OR communicat* OR interact* OR relation* OR discussion* OR round* OR meeting OR teamwork OR care))
S9	AB ((Interoccupation* or "inter occupation" or "inter occupational") N3 (collaborat* OR co-operat* OR cooperat* OR communicat* OR interact* OR relation* OR discussion* OR round* OR meeting OR teamwork OR care))
S10	TI ((transdisciplinary or "trans disciplinary") N3 (collaborat* OR co-operat* OR cooperat* OR communicat* OR interact* OR relation* OR discussion* OR round* OR meeting OR teamwork OR care))
S11	AB ((transdisciplinary or "trans disciplinary") N3 (collaborat* OR co-operat* OR cooperat* OR communicat* OR interact* OR relation* OR discussion* OR round* OR meeting OR teamwork OR care))
S12	TI (("doctor nurse" or "physician nurse") N3 (collaborat* OR co-operat* OR cooperat* OR communicat* OR interact* OR relation* OR discussion* OR round* OR meeting OR teamwork OR care))
S13	AB (("doctor nurse" or "physician nurse") N3 (collaborat* OR co-operat* OR cooperat* OR communicat* OR interact* OR relation* OR discussion* OR round* OR meeting OR teamwork OR care))
S14	S1 OR S2 OR S3 OR S4 OR S5 OR S6 OR S7 OR S8 OR S9 OR S10 OR S11 OR S12 OR S13
S15	(MH "Mortality") OR (MH "Death") OR (MH "Hospital Mortality")
S16	TI (death* or mortalit* or fatalit*)
S17	AB (death* or mortalit* or fatalit*)
S18	S15 OR S16 OR S17
S19	S14 AND S18

Cochrane database

S. No.	Search
1	MeSH descriptor: [Interprofessional Relations] explode all trees
2	MeSH descriptor: [Patient Care Planning] explode all trees
3	MeSH descriptor: [Physician-Nurse Relations] explode all trees
4	(Interprofessional or "Inter professional" or Intersectoral or interdisciplinary or "inter disciplinary") NEAR/3 (collaborat* OR co-operat* OR cooperat* OR communicat* OR interact* OR relation* OR discussion* OR round* OR meeting OR teamwork OR care):ti,ab
5	(Multiprofessional or "multi professional" or multidisciplinary or "multi disciplinary") NEAR/3 (collaborat* OR co-operat* OR cooperat* OR communicat* OR interact* OR relation* OR discussion* OR round* OR meeting OR teamwork OR care):ti,ab
6	(crossdisciplinary or "cross disciplinary") NEAR/3 (collaborat* OR co-operat* OR cooperat* OR communicat* OR interact* OR relation* OR discussion* OR round* OR meeting OR teamwork OR care):ti,ab
7	(transdisciplinary or "trans disciplinary") NEAR/3 (collaborat* OR co-operat* OR cooperat* OR communicat* OR interact* OR relation* OR discussion* OR round* OR meeting OR teamwork OR care):ti,ab
8	((Interoccupation* or "inter occupation" or "inter occupational") NEAR/3 (collaborat* OR co-operat* OR cooperat* OR communicat* OR interact* OR relation* OR discussion* OR round* OR meeting OR teamwork OR care)):ti,ab
9	("doctor nurse" OR "physician nurse") NEAR/3 (collaborat* OR co-operat* OR cooperat* OR communicat* OR interact* OR relation* OR discussion* OR round* OR meeting OR teamwork OR care):ti,ab
10	#1 or #2 or #3 or #4 or #5 or #6 or #7 or #8 or #9
11	MeSH descriptor: [Death] explode all trees
12	MeSH descriptor: [Mortality] explode all trees
13	(Death* or mortalit* or fatalit*):ti,ab
14	#11 or #12 or #13
15	#10 AND #14
