# Supplementary material for: Association between the Quantity of Nurse–Doctor Interprofessional Collaboration and in-Patient Mortality: A Systematic Review
Source: Int J Environ Res Public Health. 2024 Apr 17;21(4):494. doi: 10.3390/ijerph21040494 (PMC11050129; doi:10.3390/ijerph21040494)
Supplement: Supplementary file 1 [file ijerph-21-00494-s001.zip › Supplementary material S3_Characteristics of the measures of collaboration.pdf]

**Supplementary document 3:** Characteristics of the instruments

| Citation | Measures | Characteristics of the instrument | Validity test |
|---|---|---|---|
| Baggs 1990, Baggs et al. 1992 | DAT | The decision about transfer (DAT) tool had six items. The first two items measured collaboration and satisfaction with the transfer decision. Each item was scored 1-7 on a Likert Scale. The responses included ('1', no collaboration, '7', complete collaboration) and ('1', not satisfied, '7', fully satisfied) for the collaboration and satisfaction subunits. Items 3 and 4 measured the alternatives and need to transfer the patient from the intensive care unit. A 4-point Likert scale measured the choice's availability - 1 'disagree' to 4 'completely agree'. Item 5 measured the appropriateness of transfer of the patient as 'appropriate', 'should have been transferred earlier' or 'should have stayed longer'. Participants had an option to provide the reason, should they indicate that a patient should have stayed longer. The final item asked if he/she was responsible for the transfer of the patient. | The authors tested the face validity with ten nurses from a medical ICU. The authors acknowledged that it was not possible to measure the construct validity of the instrument.<br><br>To ensure validity and reliability of the DAT instrument, authors correlated scores with previously established tools, the Collaborative Practice Scale (CPS) and Index of Work Satisfaction (IWS).<br><br>The correlation between DAT and CPS was 0.27 (p<0.05, one tail) for nurses and 0.36 (p<0.05, one tail) for doctors. The correlation between DAT and IWS was 0.24 (p<0.05, one tail). Collaboration was associated with satisfaction about decision making for both nurses (r=0.67, p=0.000) and doctors (r=0.026, p=0.0000) |
| Baggs et al. 1999 | CSACD | The CSACD Instruments had questions to measure collaboration and satisfaction. Seven questions measured nurse-doctor collaboration. The first six questions measured collaboration attributes (plan together, communication, share decision making, cooperation, assertion, and coordination). The final question measured overall collaboration for each patient transfer. Each attribute was scored 1 (strongly disagree) to 7 (strongly agree). Two additional questions measured satisfaction; one about the satisfaction with the decision and another about the decision-making process irrespective of the decision made.<br><br>The total score for each patient transfer was between 7 to 49. The collaboration score for all patients transferred by an individual nurse/doctor was calculated. An average score for each nurse/doctor was then computed by dividing the total scores by the number of patients transferred. | Experts* (nursing and medical experts with prior publications about collaboration) (n=12), and service providers (staff nurses and residents working in a medical ICU) (n=11) reviewed the questionnaire before the validation study.<br><br>Thirty-two staff nurses and 26 paediatric residents working in a neonatal ICU participated in a reliability study. Factor loading for six items was high (0.82-0.93). The internal consistency of these six items was high (Cronbach's alpha=0.93). The global collaboration score was correlated with the sum of six attributes for collaboration (r=0.87). There was a high correlation between collaboration and satisfaction (r=0.66).<br><br>The alpha coefficient for the scores ranged from 0.9-0.96 at the three study sites. |
| Kang 2016<br>Kang et al. 2020 | PES-NWI | The nurse-physician relations subscale of the Practice Environment Scale of the Nurse working Index has three items - teamwork, good working relationship, and collaboration.<br><br>Each item is scored 1-4. An average score for all three items is calculated with a final score ranging from 1-4. | This instrument has established validity and reliability (Lake 2002).<br><br>Factor analysis for individual components were - teamwork (0.83), good working relationship (0.76), and collaboration (0.82).<br><br>The alpha coefficient for individual components- teamwork (0.782), good working relationship (0.847), and collaboration (0.796) |

* Information obtained from Baggs (1994)
