# Supplementary material for: Association between the Quantity of Nurse–Doctor Interprofessional Collaboration and in-Patient Mortality: A Systematic Review
Source: Int J Environ Res Public Health. 2024 Apr 17;21(4):494. doi: 10.3390/ijerph21040494 (PMC11050129; doi:10.3390/ijerph21040494)
Supplement: Supplementary file 1 [file ijerph-21-00494-s001.zip › Supplementary material S4_Risk of bias of the included studies.pdf]

Supplementary document 4: Risk of bias assessment

Section	Criteria	Baggs (1990)<br>Baggs et al. (1992)	Baggs et al., (1999)	Kang (2016)<br>Kang et al. (2020)
1	**Selection bias**			
1	Are the individuals selected to participate in the study likely to be representative of the target population?<br>1 Very likely 2 Somewhat likely<br>3 Not likely 4 Cannot tell	1	1	2
1	What percentage of selected individuals agreed to participate?<br>1 80 - 100% agreement 2 60 – 79% agreement<br>3 less than 60% agreement 4 Not applicable 5 Cannot tell	1	2	5
**Rate this section**<br>**Strong:** The selected individuals are very likely to be representative of the target population (Q1 is 1) and there is greater than 80% participation (Q2 is 1).<br>**Moderate:** The selected individuals are at least somewhat likely to be representative of the target population (Q1 is 1 or 2); and there is 60 - 79% participation (Q2 is 2). 'Moderate' may also be assigned if Q1 is 1 or 2 and Q2 is 5 (can't tell).<br>**Weak:** The selected individuals are not likely to be representative of the target population (Q1 is 3); or there is less than 60% participation (Q2 is 3) or selection is not described (Q1 is 4); and the level of participation is not described (Q2 is 5)		**1**	**2**	**2**
2	**Study design**			
1	Indicate the study design<br>1 Randomised controlled trial 2 Controlled clinical trial<br>3 Cohort analytic (two group pre + post)<br>4 Case-control<br>5 Cohort (one group pre + post (before and after))<br>6 Interrupted time series<br>7 Other specify __________________________ 8 Cannot tell	prospective observational	prospective observational	secondary data
2	Was the study described as randomised? (If NO, go to Component C)	No	No	No
3	If Yes, was the method of randomisation described?			
4	If Yes, was the method appropriate?			
**Rate this section**<br>**Strong:** will be assigned to those articles that described RCTs and CCTs.<br>**Moderate:** will be assigned to those that described a cohort analytic study, a case control study, a cohort design, or an interrupted time series.<br>**Weak:** will be assigned to those that used any other method or did not state the method used.		**3**	**3**	**3**
3	**Confounders**			
1	Were there important differences between groups prior to the intervention?<br>1 Yes 2 No 3 Cannot tell	1	1	1
2	If yes, indicate the percentage of relevant confounders that were controlled (either in the design (e.g. stratification, matching) or analysis)?<br>1 80 – 100% (most)<br>2 60 – 79% (some)<br>3 Less than 60% (few or none)<br>4 Cannot Tell	3	3	3
**Rate this section**<br>**Strong:** will be assigned to those articles that controlled for at least 80% of relevant confounders (Q1 is 2); or (Q2 is 1).<br>**Moderate:** will be given to those studies that controlled for 60 – 79% of relevant confounders (Q1 is 1) and (Q2 is 2).<br>**Weak:** will be assigned when less than 60% of relevant confounders were controlled (Q1 is 1) and (Q2 is 3) or control of confounders was not described (Q1 is 3) and (Q2 is 4).		**3**	**3**	**3**

4	**Blinding**			
1	Was (were) the outcome assessor(s) aware of the intervention or exposure status of participants?<br>1 Yes 2 No 3 Cannot tell	3	3	3
2	Were the study participants aware of the research question?<br>1 Yes 2 No 3 Cannot tell	3	3	3
**Rate this section**<br>**Strong:** The outcome assessor is not aware of the intervention status of participants (Q1 is 2); and the study participants are not aware of the research question (Q2 is 2).<br>**Moderate:** The outcome assessor is not aware of the intervention status of participants (Q1 is 2); or the study participants are not aware of the research question (Q2 is 2).<br>**Weak:** The outcome assessor is aware of the intervention status of participants (Q1 is 1); and the study participants are aware of the research question (Q2 is 1); or blinding is not described (Q1 is 3 and Q2 is 3)		**3**	**3**	**3**
5	**Data collection methods**			
1	Were data collection tools shown to be valid?<br>1 Yes 2 No 3 Cannot tell	1	1	1
2	Were data collection tools shown to be reliable?<br>1 Yes 2 No 3 Cannot tell	1	1	1
**Rate this section**<br>**Strong:** The data collection tools have been shown to be valid (Q1 is 1); and the data collection tools have been shown to be reliable (Q2 is 1).<br>**Moderate:** The data collection tools have been shown to be valid (Q1 is 1); and the data collection tools have not been shown to be reliable (Q2 is 2) or reliability is not described (Q2 is 3).<br>**Weak:** The data collection tools have not been shown to be valid (Q1 is 2) or both reliability and validity are not described (Q1 is 3 and Q2 is 3)**.**		**1**	**1**	**1**
6	**Withdrawals and drop-outs**			
1	Were withdrawals and drop-outs reported in terms of numbers and/or reasons per group?<br>1 Yes 2 No 3 Cannot tell<br>4 Not Applicable (i.e. one-time surveys or interviews)	4	4	4
2	Indicate the percentage of participants completing the study. (If the percentage differs by groups, record the lowest).<br>1 80 -100% 2 60 - 79% 3 < 60% 4 Cannot tell 5<br>Not Applicable (i.e. Retrospective case-control)	5	5	5
**Rate this section**<br>**Strong:** will be assigned when the follow-up rate is 80% or greater (Q1 is 1 and Q2 is 1).<br>**Moderate:** will be assigned when the follow-up rate is 60 – 79% (Q2 is 2) OR Q1 is 4 or Q2 is 5.<br>**Weak:** will be assigned when a follow-up rate is less than 60% (Q2 is 3) or if the withdrawals and drop-outs were not described (Q1 is No or Q2 is 4).<br>**Not Applicable:** if Q1 is 4 or Q2 is 5.		**N/A**	**N/A**	**N/A**
7	**Intervention integrity**			
1	What percentage of participants received the allocated intervention or exposure of interest?<br>1 80 -100% 2 60 - 79% 3 < 60% 4 Cannot tell	4	4	4
2	Is it likely that subjects received an unintended intervention (contamination or co-intervention) that may influence the results?<br>1 Yes 2 No 3 Cannot tell	3	3	3
8	**Analysis**			
1	Indicate the unit of allocation<br>1. community<br>2. organisation/institution<br>3. practice/office<br>4. individual	individual	individual	individual

2	Indicate the unit of analysis<br>1. community<br>2. organisation/institution<br>3. practice/office<br>4. individual	individual	individual	organization/institution
3	Are the statistical methods appropriate for the study design?<br>1 Yes 2 No 3 Cannot tell	1	1	1
4	Is the analysis performed by intervention allocation status (i.e., intention to treat) rather than the actual intervention received?<br>1 Yes 2 No 3 Cannot tell	N/A	N/A	N/A
	**Global Rating**			
1	**Selection bias**<br>1. Strong 2. Moderate 3. Weak	1	2	2
2	**Study design**<br>1. Strong 2. Moderate 3. Weak	3	3	3
3	**Confounders**<br>1. Strong 2. Moderate 3. Weak	3	3	3
4	**Blinding**<br>1. Strong 2. Moderate 3. Weak	3	3	3
5	**Data collection method**<br>1. Strong 2. Moderate 3. Weak	1	1	1
6	**Withdrawals and dropouts**<br>1. Strong 2. Moderate 3. Weak	N/A	N/A	N/A
	**Global rating for this paper**<br>1 Strong (no WEAK ratings)<br>2 Moderate (one WEAK rating)<br>3 Weak (two or more WEAK ratings)	**Weak**	**Weak**	**Weak**
